# Mediterranean diet lowers all-cause and cardiovascular mortality for patients with metabolic syndrome

**DOI:** 10.1186/s13098-023-01052-7

**Published:** 2023-05-23

**Authors:** Hongxuan Fan, Yongle Wang, Zhaoyu Ren, Xuchang Liu, Jianqi Zhao, Yalin Yuan, Xiaoning Fei, Xiaosu Song, Fengqin Wang, Bin Liang

**Affiliations:** 1 Department of Cardiology, The Second Hospital of Shanxi Medical University, 382 Wuyi Road, Taiyuan, 030001 Shanxi China; 2 Department of Neurology, The First Hospital of Shanxi Medical University, 85 Jiefang South Road, Taiyuan, 030001 Shanxi China; 3 Department of Urology, The First Hospital of Shanxi Medical University, 85 Jiefang South Road, Taiyuan, 030001 Shanxi China; 4 Shanxi Medical University, No. 56, Xinjian South Road, Taiyuan, 030001 Shanxi China; 5 Department of Cardiology, Yangquan First People’s Hospital, No. 167, South Street, Yangquan City, 030001 Shanxi China

## Abstract

A Mediterranean-style diet (MED) can promote people lengthen the span of life and avoid atherosclerotic cardiovascular disease (ASCVD) in primary prevention. Metabolic syndrome (MetS) can significantly reduce life expectancy and increase the risk of ASCVD. However, few studies have focused on the role of the Mediterranean diet in patients with MetS. Participants in the National Health and Nutrition Examination Survey (NHANES) with MetS (N = 8301) from 2007 to 2018 were examined. A 9-point evaluation scorewas used to measure the degree of adherence to the MED diet. In order to compare the various levels of adherence to the MED diet and the effects of the specific MED diet components on all-cause and cardiovascular mortality, Cox regression models were utilized. Among the 8301 participants with MetS, about 13.0% (1080 of 8301) died after a median follow-up of 6.3 years. In this study, participants with MetS with adherence to high-quality and moderate-quality Mediterranean diet were significantly associated with lower all-cause mortality as well as cardiovascular mortality during the follow-up period. Futhermore, in joint analysis of the Mediterranean diet and sedentary behavior or depression, we found that high-quality or moderate-quality Mediterranean diet could attenuate, even reverse the adverse effects of sedentary behavior and depression on all-cause and cardiovascular mortality in participants with MetS. Among the components of the MED diet, greater intakes of vegetables, legumes, nuts and high MUFA/SFA ratio were significantly associated with lower all-cause mortality and greater vegetables intake was significantly associated with lower cardiovascular mortality, while more red/processed meat intake was significantly associated with higher cardiovascular mortality in participants with MetS.

## Introduction

MetS is defined as the presence of central obesity combined with at least two of the following four conditions: hypertension, diabetes mellitus, hyperlipidemia, and hypo-high-density lipoprotein cholesterolemia. The incidence of Mets has sharply increased as people’s living standards have improved, modern urban lifestyles have emerged, and various influences like diet, exercise, genetics, and race have become more important [1]. Prior research according to National Health and Nutrition Examination Survey (NHANES) found a prevalence of 33% in American adults, which was consistent from 2007 to 2012 [2]. And a recent study shows that the incidence of Mets is even on the rise in the U.S. from 2011–2016 [3]. Numerous clinical scientists and researchers have focused on the MetS since it was originally introduced, furthering our understanding of it. The concept of insulin resistance (IR) was first put up by Reaven [4], who noted that it plays a significant role in the emergence of all cardiovascular illnesses and is not just evident in type 2 diabetes. After discovering that IR deficiency alone could not account for the onset of cardiovascular disease in individuals of all body types, researchers turned their attention to visceral fat, which was discovered to have a positive correlation with IR and blood lipid levels in both overweight and obese populations [5]. Since the widespread use of Magnetic Resonance Imaging (MRI), it has been clear that the liver, a key component of human metabolism, might be the primary culprit in the buildup of visceral fat [6]. It is widely recognized that cardiovascular disease and death have a tight relationship with MetS [7]. Numerous researchers revealed, at the mechanistic level, that visceral fat can trigger chronic inflammatory pathways by releasing AngII, which causes oxidative stress, inflammatory factors, adiponectin, free fatty acids, disrupts human hormones, and IR [8–10]. IR, which is characterized as a decreased biological response to circulating serum insulin, is a fundamental flaw in type 2 diabetes and is also heavily linked to a variety of diseases based on it, including non-alcoholic fatty liver disease, cognitive decline, endothelial dysfunction, coronary atherosclerotic heart disease, chronic kidney disease, and various cancers, all of which increase mortality.

Since Trichopoulou et al. demonstrated in a Greek population that the Mediterranean diet prolongs life expectancy [11], there has been a steady stream of research on the Mediterranean diet. The Mediterranean diet is a commonly used to define the foods consumed in southern European nations along the Mediterranean Sea, such as Greece, Spain, France, and southern Italy, that include vegetables, fruits, fish, grains, legumes, and olive oil. The term “Mediterranean diet” is now also used to describe a straightforward, wholesome, and healthful diet. A growing body of research has shown that the nutrients in the Mediterranean diet can have anti-inflammatory effects. When compared to other dietary regimens, the Mediterranean diet (MedDiet), which emphasises the use of extra-virgin olive oil (EVOO), almonds, red wine, vegetables, and other polyphenol-rich foods, has been shown to be associated with a higher improvement in IR in obese people [12]. Studies have found that the Mediterranean diet can lower the risk of coronary heart disease, protect the brain from blood vessel damage, lower the risk of stroke, and prevent memory loss [13]. According to a recent study, people with coronary heart disease or stroke who ate a healthier Mediterranean diet had a significantly lower all-cause mortality [14].

The development of MetS is closely related to obesity. IR and its associated metabolic issues might occur as a result of the uneven production of pro- and anti-inflammatory adipocytokines, which may be treated with weight-loss regimens. Obesity is the primary characteristic of those who have MetS, and its pathological process includes visceral fat infiltration and IR. Numerous research has been done to demonstrate that weight loss and calorie restriction are efficient ways to combat MetS and lessen IR. However current research indicates that not only eating less can help lower IR, eating healthfully, such as combining with a Mediterranean diet, can have an even greater impact on reducing insulin sensitivity. Low-carbohydrate and Mediterranean diets might be superior choices to low-fat diets. The better results on lipids (with the low-carbohydrate diet) and glycemic control (with the Mediterranean diet) imply that dietary interventions may be individually tailored based on preferences and metabolic factors [15]. According to a recent study, the Mediterranean diet was more effective than the low-fat diet at preventing major cardiovascular events in secondary prevention and support the use of the Mediterranean diet in secondary prevention which could be applicable to clinical practice [16].

The beneficial effects of the Mediterranean diet on life expectancy and as a primary prevention of ASCVD have been well recognized. However, few studies have investigated the association between the Mediterranean diet and all-cause mortality and cardiovascular mortality in people with MetS. Moreover, the joint effect of Mediterranean diet and unhealthy lifestyle habits or mental diseases, such as sedentary behavior and depression, on all-cause mortality and cardiovascular mortality in people with MetS has not been studied. Lastly, the effect of specific components of the Mediterranean diet on mortality has not been analyzed in more depth. Our aim in this study was to assess the effect of the Mediterranean diet on all-cause and cardiovascular mortality in participants diagnosed with MetS from NHANES cohort, then to analyze in depth the joint effect between the Mediterranean diet and other adverse factors in daily life and to explore the impact of specific components of the Mediterranean diet on all-cause mortality and cardiovascular mortality in participants with MetS.

## Materials and methods

### Study design and patient information

A prospective cohort study was designed using the population data from The National Health and Nutrition Examination Survey (NHANES) 2007–2018 database to explore the role of Mediterranean diet on all-cause mortality and cardiovascular mortality in people with MetS, study in depth the joint effect of Mediterranean diet, sedentary behavior and depression on all-cause mortality and cardiovascular mortality in people with MetS and figure out the impact of each Mediterranean dietary component on all-cause mortality and cardiovascular mortality in people with MetS. The related data were collected from NHANES 2007–2018, and the mortality follow-up data was from the date of survey participation through December 31, 2019. The National Health and Nutrition Examination Survey (NHANES) is a collection of cross-sectional studies that are nationally representative and aimed at tracking the health of the American population. Its goal was to evaluate both the nutritional and physical health of adults and children. For in-home interviews and visits at a mobile examination center, participants are chosen from the non-institutionalized, general population of the United States using a complicated, stratified, and multi-stage probability cluster sampling approach. All survey participants supplied informed written agreement, which was approved ethically by the Ethics Review Board of the National Center for Health Statistics. Detailed questionnaires regarding dietary components, previous medical history of participants and the survival information for these populations is available are available. It is accessible to obtain this data collection for free online at https://www.cdc.gov/nchs/nhanes/index.htm.

### Definition of metabolic syndrome

All participants are selected from the NHANES 2007–2018. We characterized MetS according to International Diabetes Federation as central obesity (waist circumference, WC 102 cm in men and 88 cm in women; if BMI > 30, central obesity can be inferred and WC did not need to be evaluated) plus any two of the following four factors: Reduced HDL-C (40 mg/dL (1.03 mmol/L) in men and 50 mg/dL (1.29 mmol/L) in women) or specific treatment for this lipid abnormality; Raised fasting TGs ≥ 150 mg/dL (1.7 mmol/L) or specific treatment for this lipid abnormality; Raised blood pressure (systolic > 130 or diastolic > 85 mm Hg) or treatment of previously diagnosed hypertension and raised fasting plasma glucose (FPG) > 100 mg/dL (5.6 mmol/L) or previously diagnosed [17]. The results of the examination and laboratory test comprising the above criteria were obtained from the NHANES database. Data of body measurement included height, weight, and WC and body mass index (BMI) = weight (kg)/(height (m))^2^ can be obtained from the database. The patient rested in a sitting position for five minutes before having their blood pressure taken three times, with the mean of the three readings being used for analysis. Trained healthcare professionals used a calibrated Omron IntelliSense Blood Pressure Monitor (Model: HEM-907XL) to measure the patient’s systolic and diastolic pressures. Participants’ blood was drawn to measure their blood glucose levels after an overnight fast using a Cobas C Chemistry Analyzer (C311, Roche CO., USA). A Cobas Chemistry Analyzer was used to measure the blood’s levels of TG and HDL-C. (6000, Roche CO., USA). The NHANES survey was used to gather data on prescription medicine usage. In order to further enhance the universality of this design and make the conclusions more reliable, we additionally decided to adopt another diagnostic standard for MetS to form the new MetS participants for research. The new diagnostic criteria is that three of the above five conditions are met, which can be diagnosed as MetS.

### Definition of mediterranean diet

The dietary data of this study is derived from The United States Department of Agriculture’s Food Patterns Equivalents Database, which was applied to NHANES database. The relationship between a high-quality Mediterranean diet and obesity in the population has been previously well summarized by a relevant scale. In a group of people at high cardiovascular risk, a quick 14-item test was able to capture a substantial monotonic inverse correlation between adherence to a high-quality dietary pattern (Mediterranean diet) and obesity indices [18]. However, this scale is not fully applicable to the dietary data in the NHANES database. The alternative Mediterranean diet index (aMED) was used to calculate and measure adherence to the Mediterranean diet in this study [19].Overall intakes of fruits, vegetables, whole grains, legumes, nuts, fish, red and processed meat, the ratio of monounsaturated to saturated fat, and alcohol were assessed in order to determine the aMED. With the exception of red and processed meat, and alcohol, participants whose intake exceeded the study cohort’s median were given one point. One point was given for moderate alcohol consumption (10-25 g/day for men and 5-15 g/day for women) and for those who consumed less meat than the cohort’s median amount for both red and processed meat. Participants received a score of zero if these requirements were not met. The aMED score, which ranges from 0 to 9, thus indicates how closely a person adheres to the Mediterranean diet [14]. All dietary data are dated from 2007 to 2018, which presents six cycles of NHANES.

### Definition of physical activity and sedentary behavior

Participants in NHANES used the Global Physical Activity Questionnaire to self-report their PA data (GPAQ). It is an approved tool for PA surveillance [20]. Participants who engaged in at least 150 min per week of moderate-intensity aerobic physical activity (MPA) or 75 min per week of vigorous-intensity aerobic physical activity, or an equivalent combination of moderate and vigorous PA (1 min of VPA is equivalent to 2 min of MPA), totaling at least 150 min per week, were defined as meeting the guidelines by the World Health Organization (WHO) Guidelines on Physical Activity and Sedentary Behavior [21]. Participants were categorized as having insufficient moderate-to-vigorous work activity (MVWA) (150 min/week), insufficient moderate-to-vigorous recreational activity (MVRA) (150 min/week), sufficient MVWA (150 min/week), and sufficient MVRA (150 min/week) based on the reported number of days and time in minutes spent on moderate or vigorous work activity, as well as moderate or vigorous recreational activity. Additionally, based on the self-reported amount of time typically spent sitting during a typical day, we divided the time spent engaging in sedentary behavior into two groups (480 min/day and 480 min/day) [22]. Physical activity and sedentary behavior data were obtained from NHANES 2007–2018.

### Definition of depression

During the face-to-face MEC interview, the PHQ-9 was given to evaluate depressive symptoms over the previous two weeks [23]. The following symptoms were listed for respondents to rate on a scale of 0 to 3: anhedonia, depressed mood, sleep disturbance, fatigue, appetite changes, low self-esteem, concentration issues, psychomotor disturbances, and suicidal ideation. Total scores can range from 0 to 27, with scores below 10 denoting symptoms of depression that are clinically significant. Additionally, the PHQ-9 is a validated tool for assessing the severity of depressive symptoms (total score 1–4: minimal depression, 5–9: mild depression, 10–14: moderate depression, 15–19: moderately severe depression, and 20–27: severe depression). For detecting major depression disorder cases, a meta-analysis study suggested PHQ-9 exhibits high internal consistency and good sensitivity and specificity [24]. All PHQ-9 scale data was collected from NHANES 2007–2018.

### The collection of survival data

The National Center for Health Statistics (NCHS) has linked data collected from several NCHS population surveys with death certificate records from the National Death Index (NDI). The definition of all-cause mortality included all kinds of deaths. The sub-classifications of causes of death into cardiovascular, cancer, respiratory disease, diabetes mellitus, or miscellaneous other causes related mortality were also derived from National Death Index data (coding by International Classification of Diseases). To complement the restricted-use files and increase data access, NCHS developed public-use versions of the LMF for the 1999–2018 National Health and Nutrition Examination Survey (NHANES). Information regarding vital status was not perturbed. The public-use LMF provide mortality follow-up data from the date of survey participation through December 31, 2019.

### Other covariates

Demographic variables included age, sex, race, education, marital status and poverty to income ratio. Race/ethnicity were categorized as Hispanic-Mexican American, Hispanic-Other Hispanic, Non-Hispanic Black, Non-Hispanic White and other (including multiple races). Education was categorized as less than 9th grade, 9-11th grade (includes 12th grade with no diploma), high school Grad/GED or equivalent, some college or AA degree and college graduate or above. Marital status was categorized as married, widowed, divorced, separated, never married and living with partner. PIR was presented a ratio of family income to poverty. Smoking status was defined as former or current smokers if they smoked at least 100 cigarettes in one’s lifetime. Cardiovascular disease is defined as having been diagnosed with any of the following four diseases: congestive heart failure, coronary artery disease, angina pectoris, or heart attack. Treatment for hypertension was defined as having taken prescription for hypertension. Treatment for diabetes was defined as having taken insulin. Treatment for cholesterol was defined as having taken prescription for cholesterol.

### Statistical analysis

We adopted the NHANES official recommended statistics due to the complexity of the surveys used in the NHANES study. In order to accurately reflect the population of the United States, all analyses needed to be properly weighted. The weighted estimates were calculated in accordance with the analytical guidelines from the National Health and Nutrition Examination Survey for the years 2007 through 2018, which is accessible online. All data were analyzed using Empower Statistics version 4.1, GraphPad Prism 8.0 (San Diego, California, USA) and Free Statistics version 1.7 (Beijing, China). In Table 1, continuous variables were presented as survey-weighted mean (95% CI), while categorical variables were presented as survey-weighted percentage (95% CI). For continuous variables, we examined for significant difference in baseline characteristics between three aMED subgroups using the analysis of variance (ANOVA) test. The test for trend was performed with a polynominal contrast procedure. Using Bonferroni corrections, comparisons between two different groups were made. For categorical variables, we used Chi-square test to examine significant difference between different groups. The test for trend was performed using Trend Chi-square test. We used the weighted Cox proportional hazards regression models of the SURVEYPHREG Procedure with adjustment for relevant variables to compare the hazard ratio (HR) and 95% confidence interval (CI) for the associations of MED diet and influences of its individual food components on all-cause and cardiovascular mortality. The covariables adjusted in Cox regression analysis were age, sex, race, education, marital status, poverty to income ratio, cardiovascular disease, smoking, treatment for hypertension, treatment for diabetes and treatment for cholesterol. In all of the statistical analyses, significance was set at a two-sided *P* < 0.05.

## Results

### Clinical and biochemical characteristics in NHANES participants with MetS in different Mediterranean diet score groups

**Fig. 1:**
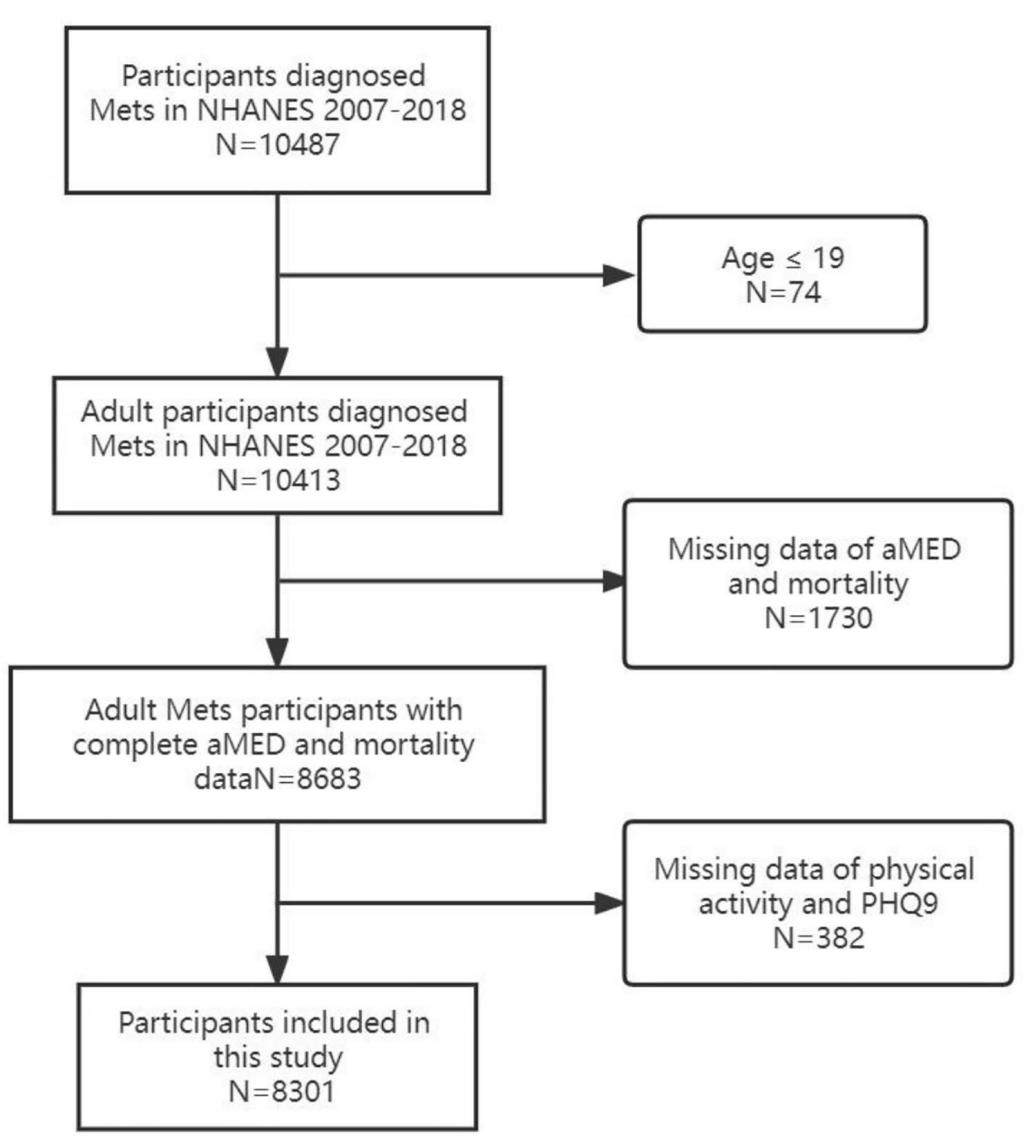
Flowchart of NHANES participants with Mets enrolled in this study

For our study, six cycles of continuous NHANES 2007–2018 data were employed, with 10,487 participants diagnosed Mets included. Individuals aged < 20 years and without complete data of diet, physical activity, PHQ9 and mortality were excluded, resulting in 8301 subjects included in our analysis. Figure 1 illustrates the process of participant inclusion.

Participants in the National Health and Nutrition Examination Survey (NHANES) with Mets (N = 8301) from 2007 to 2018 were examined. Up to the end of 2019, their unique vital status was connected to the National Death Index. After a median follow-up of 6.3 years, 1080 out of the 8301(about 13.0%) individuals with MetS died. They were categorized into below median (aMED scores 0–3), median (aMED scores 4), and above median (aMED scores 5–9) groups based on their aMED scores. Table 1 suggested participants in the above median aMED (scores 5–9) appeared to be older, had a considerably smaller waist circumference, and have higher HDL levels. These participants in the aMED (scores 5–9) had diets higher in MUFA/SFA, lower in meat and higher in vegetables, fruits, whole grains, legumes, nuts and seafood. This group had a lower percentage of depressive symptoms and a higher percentage of recreational activities. According to another diagnostic criterion of MetS, we finally enrolled 9302 MetS participants from 2007 to 2018 in NHANES. After a median follow-up of 6.2 years, 1274 out of the 9302(about 13.7%) individuals with MetS died. They were also categorized into 3 groups. There are 4084, 2118 and 3100 MetS participants in Below median (aMED scores 0–3) group, median (aMED scores 4) and above median (aMED scores 5–9) groups, respectively.

**Table 1:** Baseline demographics of NHANES participants with MetS in different Mediterranean diet score groups

	Total	aMED (Below Median)	aMED (Median)	aMED (Above Median)	P1	P2	P for trend
		(Score 0–3)	(Score 4)	(Score 5–9)			
Number of subjects	8301	3705	1887	2709			
Age, year	56.7 (56.2 ,57.3)	55.6 (54.9 ,56.4)	56.7 (55.6 ,57.8)	58.2 (57.5 ,59.0)	0.0669	< 0.0001	< 0.0001
Female, %	55.1 (53.2 ,57.0)	56.4 (53.9 ,58.9)	53.6 (50.1 ,57.0)	54.4 (50.8 ,57.9)	0.1985	0.3317	0.2969
Race (Mexico), %	7.9 (6.5 ,9.6)	6.2 (5.0 ,7.8)	9.1 (7.0 ,11.7)	9.5 (7.7 ,11.7)	0.0008	< 0.0001	< 0.0001
Education (College Graduate or above), %	23.7 (21.6 ,25.9)	17.8 (15.3 ,20.7)	22.2 (19.1 ,25.6)	32.8 (29.9 ,35.8)	0.0161	< 0.0001	< 0.0001
Marital status (Married), %	58.9 (56.8 ,61.0)	54.7 (52.1 ,57.2)	60.3 (56.4 ,64.1)	63.8 (60.1 ,67.3)	0.0124	0.0001	0.0001
Poverty to income ratio	2.9 (2.9 ,3.0)	2.7 (2.5 ,2.8)	2.9 (2.8 ,3.1)	3.4 (3.2 ,3.5)	0.0042	< 0.0001	< 0.0001
Waist, cm	112.2 (111.7 ,112.8)	113.0 (112.2 ,113.8)	112.0 (110.9 ,113.1)	111.4 (110.5 ,112.3)	0.1183	0.0072	0.0063
BMI	33.9 (33.6 ,34.1)	34.2 (33.8 ,34.6)	33.8 (33.2 ,34.3)	33.5 (33.0 ,33.9)	0.204	0.0183	0.0173
SBP, mmHg	125.8 (124.5 ,127.1)	125.3 (123.7 ,126.9)	127.6 (125.9 ,129.2)	125.3 (123.2 ,127.4)	0.0357	0.9954	0.871
DBP, mmHg	70.9 (70.4 ,71.5)	70.5 (69.6 ,71.3)	71.9 (70.9 ,72.9)	70.9 (70.0 ,71.8)	0.0114	0.4393	0.3503
Triglyceride, mmol/L	1.8 (1.8 ,1.9)	1.8 (1.8 ,1.9)	1.8 (1.7 ,1.9)	1.9 (1.8 ,2.0)	0.3427	0.672	0.7349
Triglyceride, mg/dL	163.2 (158.1 ,168.4)	163.6 (157.3 ,170.0)	158.1 (148.5 ,167.7)	166.2 (155.8 ,176.6)	0.3429	0.6718	0.7347
HDL-C, mmol/L	1.2 (1.2 ,1.2)	1.2 (1.2 ,1.2)	1.2 (1.2 ,1.3)	1.2 (1.2 ,1.3)	0.0195	0.0002	0.0002
HDL-C, mg/dL	46.9 (46.3 ,47.5)	45.8 (45.0 ,46.5)	47.3 (46.2 ,48.3)	48.2 (47.2 ,49.3)	0.02	0.0002	0.0002
Fasting Glucose, mmol/L	6.6 (6.5 ,6.7)	6.6 (6.5 ,6.8)	6.8 (6.5 ,7.1)	6.6 (6.4 ,6.7)	0.2538	0.6841	0.8019
Fasting Glucose, mg/dL	119.8 (118.0 ,121.6)	119.3 (116.8 ,121.8)	122.5 (117.7 ,127.3)	118.5 (115.9 ,121.2)	0.2532	0.6868	0.8048
Vegetables, cup	1.1 (1.0 ,1.1)	0.8 (0.7 ,0.8)	1.1 (1.1 ,1.2)	1.4 (1.4 ,1.5)	< 0.0001	< 0.0001	< 0.0001
Fruits, cup	0.9 (0.9 ,1.0)	0.6 (0.5 ,0.6)	1.0 (0.9 ,1.1)	1.3 (1.3 ,1.4)	< 0.0001	< 0.0001	< 0.0001
Grain, ounce	6.2 (6.1 ,6.3)	5.4 (5.2 ,5.6)	6.5 (6.2 ,6.7)	7.1 (6.9 ,7.4)	< 0.0001	< 0.0001	< 0.0001
Meat, ounce	2.7 (2.6 ,2.8)	3.1 (2.9 ,3.2)	2.8 (2.6 ,3.0)	2.2 (2.0 ,2.3)	0.0051	< 0.0001	< 0.0001
Legumes, ounce	0.6 (0.5 ,0.6)	0.2 (0.2 ,0.3)	0.6 (0.5 ,0.7)	1.0 (0.9 ,1.1)	< 0.0001	< 0.0001	< 0.0001
Seafood, ounce	0.6 (0.6 ,0.7)	0.2 (0.2 ,0.3)	0.6 (0.6 ,0.7)	1.2 (1.1 ,1.3)	< 0.0001	< 0.0001	< 0.0001
Nuts, ounce	0.7 (0.6 ,0.7)	0.3 (0.2 ,0.3)	0.7 (0.6 ,0.7)	1.2 (1.1 ,1.4)	< 0.0001	< 0.0001	< 0.0001
MUFA/SFA	1.1 (1.1 ,1.1)	1.0 (1.0 ,1.0)	1.1 (1.1 ,1.1)	1.3 (1.2 ,1.3)	< 0.0001	< 0.0001	< 0.0001
Alcohol, ounce	0.5 (0.4 ,0.5)	0.5 (0.4 ,0.6)	0.5 (0.4 ,0.6)	0.5 (0.4 ,0.6)	0.771	0.9778	0.9953
Alcohol, gram	6.8 (6.1 ,7.4)	6.8 (5.8 ,7.9)	6.6 (5.1 ,8.1)	6.9 (5.7 ,8.0)	0.771	0.9778	0.9953
Depression, %	10.3 (9.3 ,11.4)	12.3 (10.8 ,13.9)	10.4 (8.1 ,13.1)	7.6 (6.0 ,9.6)	0.2293	0.0002	0.0001
Sedentary behavior, %	39.0 (37.3 ,40.8)	39.9 (37.4 ,42.5)	36.9 (33.2 ,40.7)	39.3 (36.6 ,42.1)	0.2022	0.727	0.6524
Physical activity, %	49.9 (47.9 ,51.8)	48.6 (45.8 ,51.3)	50.1 (45.9 ,54.2)	51.5 (48.3 ,54.8)	0.5502	0.1508	0.1497
Work activity, %	35.7 (33.8 ,37.6)	36.7 (34.1 ,39.3)	36.7 (32.9 ,40.7)	33.6 (30.1 ,37.2)	0.9946	0.1356	0.1518
Recreation activity, %	25.2 (23.5 ,26.9)	20.9 (18.7 ,23.1)	26.5 (23.5 ,29.9)	30.1 (27.4 ,33.1)	0.0022	< 0.0001	< 0.0001

Data are presented as mean (95% confidence interval) or n (%);

P1, comparation between aMED median and aMED below median;

P2, comparation between aMED above median and aMED below median;

P for trend based on variable containing mean value for each group

### Cox regression analysis of factors associated with all-cause and cardiovascular mortality in MetS participants

**Fig. 2:**
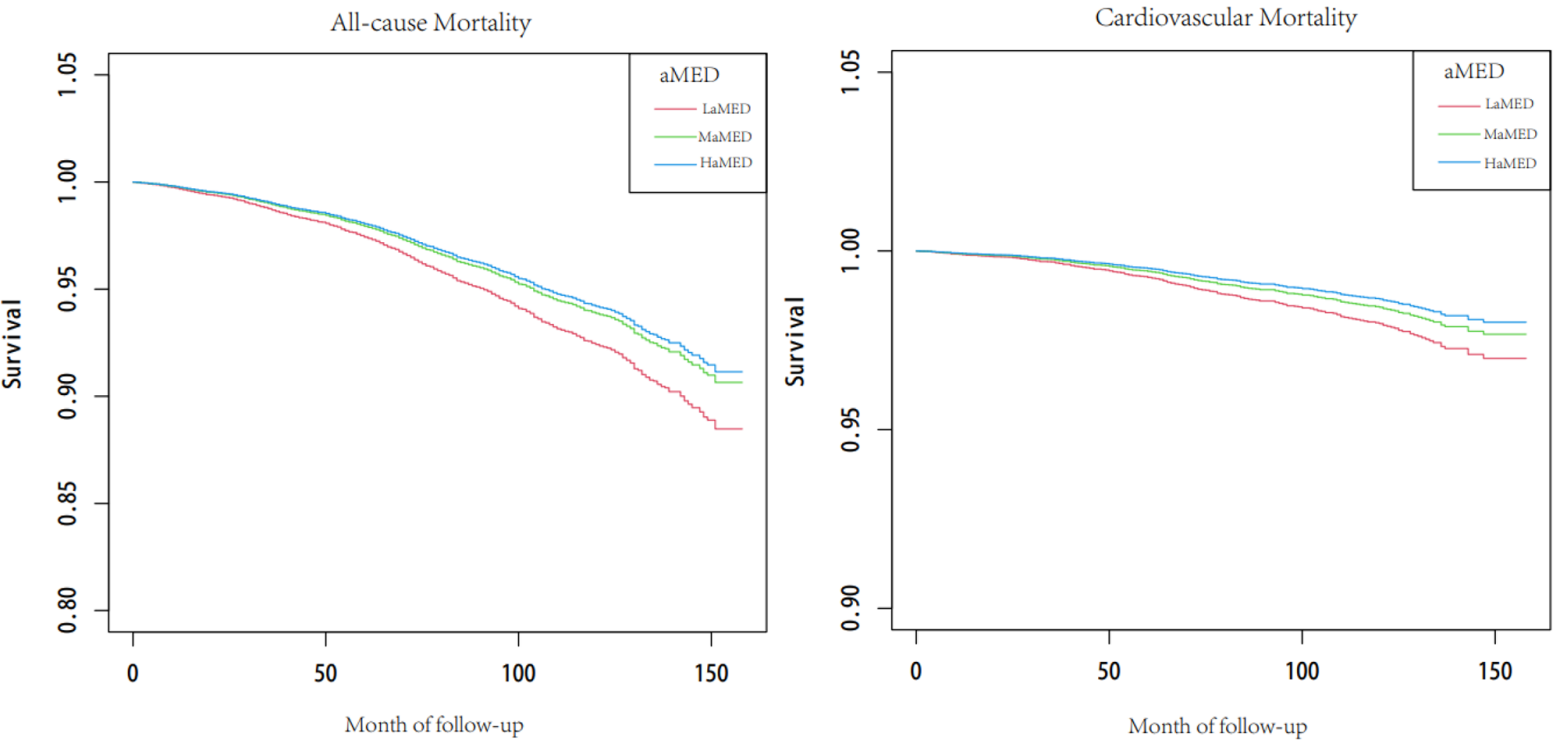
Survival curves of different dietary classes with all-cause mortality and cardiovascular mortality, respectively. aMED: alternative Mediterranean diet score, LaMED: below median alternative Mediterranean diet score, MaMED: median alternative Mediterranean diet score, HaMED: above median alternative Mediterranean diet score. Model was adjusted for age, sex, race, education, marital status, poverty to income ratio, cardiovascular disease and smoking

To explore independent predictors strongly associated with all-cause mortality and cardiovascular mortality of MetS participants, the cox regression analysis was performed. The cox regression analysis showed that alternative Mediterranean diet score (Median and Above Median), physical activity, work activity, recreation activity, treatment for hypertension and treatment for diabetes were significantly associated with a lower HR for all-cause mortality while depression, sedentary behavior, smoking, cardiovascular disease were significantly associated with a higher HR for all-cause mortality after adjustment for age, sex, race, education, marital status and poverty to income ratio. The cox regression analysis also showed that alternative Mediterranean diet score (Median and Above Median), recreation activity and treatment for diabetes were significantly associated with a lower HR for cardiovascular mortality whereas sedentary behavior and cardiovascular disease were significantly associated with a higher HR for cardiovascular mortality after adjustment for age, sex, race, education, marital status and poverty to income ratio. These results are presented in Table 2. Figure 2 shows the survival curves of different dietary classes with all-cause death and cardiovascular death after adjustment for age, sex, race, education, marital status and poverty to income ratio, cardiovascular disease and smoking, respectively.

**Table 2:** Factors associated with all-cause and cardiovascular mortality in MetS participants

	All-cause Mortality			Cardiovascular Mortality		
	HR (95%CI) *P*			HR (95%CI) *P*		
aMED (Below Median)	Ref.			Ref.		
aMED (Median)	0.77	0.65–0.90	0.00	0.71	0.52–0.98	0.03
aMED (Above Median)	0.74	0.64–0.86	0.00.	0.65	0.49–0.87	0.00
Depression	1.48	1.22–1.79	0.00	1.41	0.97–2.06	0.07
Sedentary behavior	1.79	1.57–2.03	0.00	2.02	1.59–2.57	0.00
Physical activity	0.68	0.59–0.78	0.00	0.79	0.60–1.02	0.07
Work activity	0.70	0.59–0.82	0.00	0.90	0.67–1.20	0.48
Recreation activity	0.63	0.52–0.75	0.00	0.63	0.45–0.90	0.01
Smoking	1.50	1.31–1.71	0.00	1.27	0.98–1.63	0.07
Cardiovascular disease	1.83	1.60–2.10	0.00	3.03	2.36–3.88	0.00
Treatment for HTN	0.42	0.22–0.82	0.01	0.34	0.08–1.39	0.13
Treatment for diabetes	0.48	0.41–0.57	0.00	0.48	0.35–0.66	0.00
Treatment for cholesterol	1.02	0.84–1.23	0.83	1.18	0.84–1.66	0.35

Model was adjusted for age, sex, race, education, marital status and poverty to income ratio

### Cox regression analysis of participants with MetS for all-cause and cardiovascular mortality in different dietary classes with and without sedentary behavior

**Fig. 3:**
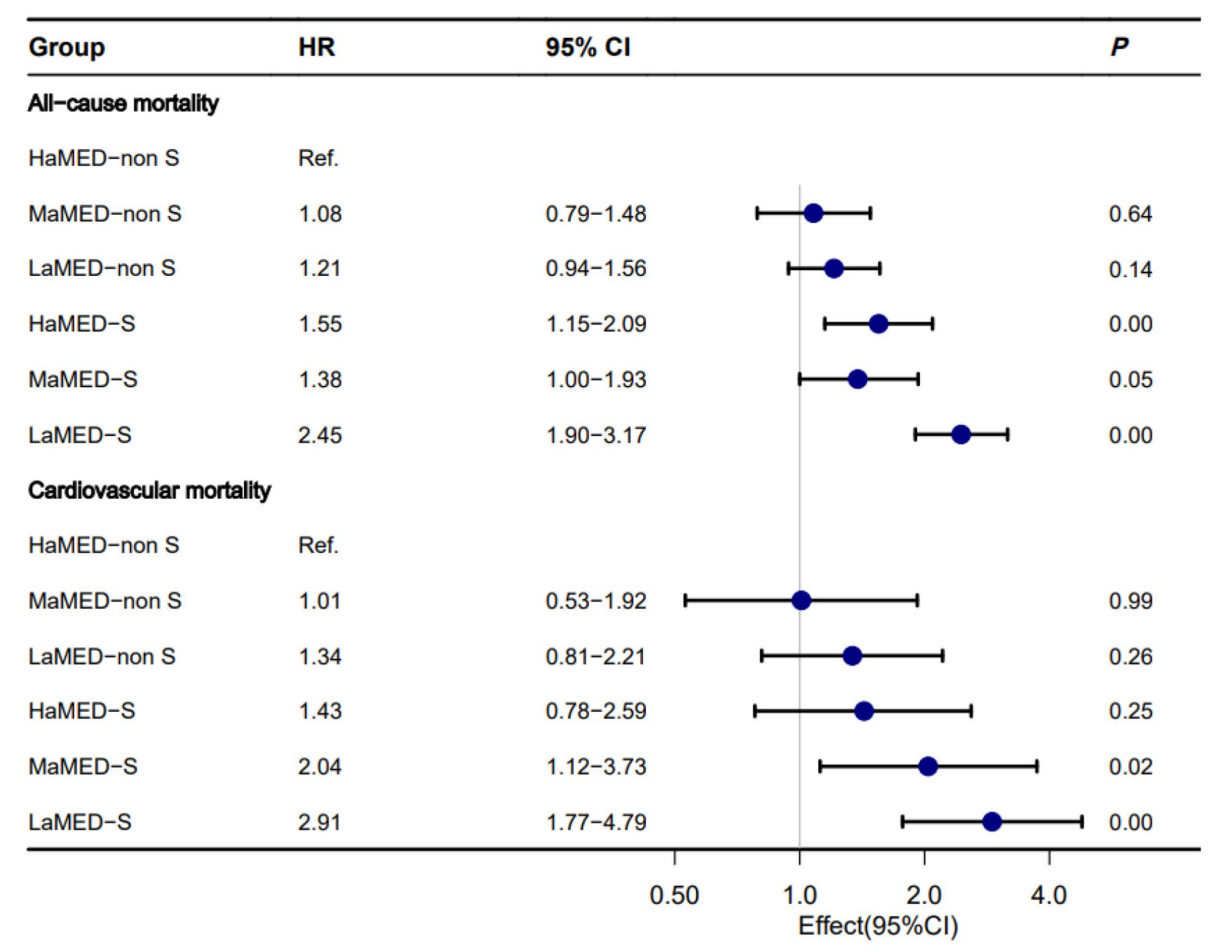
Forest plot of cox regression analysis of participants with MetS for all-cause and cardiovascular mortality according to aMED and sedentary behavior

To investigate the joint effect of the Mediterranean diet and sedentary behavior on all-cause mortality and cardiovascular mortality in participants with MetS, we divided all paticipants with MetS into the following six groups: HaMED-non S (MetS participants with above median aMED without sedentary behavior), MaMED-non S (MetS participants with median aMED without sedentary behavior), LaMED-non S (MetS participants with below median aMED without sedentary behavior), HaMED-S (MetS participants with above median aMED with sedentary behavior), MaMED-S (MetS participants with median aMED with sedentary behavior) and LaMED-S (MetS participants with below median aMED with sedentary behavior). Cox regression analysis of the all-cause mortality risk for MaMED-non S, LaMED-non S, HaMED-S, MaMED-S, LaMED-S groups using HaMED-non S group as the control group showed that, after the adjustment for age, sex, race, education, marital status, poverty to income ratio, cardiovascular disease, smoking, treatment for hypertension, treatment for diabetes and treatment for cholesterol, there was no significant difference in HR between HaMED-non S, MaMED-non S and LaMED-non S groups. HaMED-S and MaMED-S groups showed a similar increased HR in all-cause mortality (HR, 1.55; 95% CI, 1.15–2.09; P = 0.00), (HR, 1.38; 95% CI, 1.00–1.93; P = 0.05) compared to HaMED-non S group. While LaMED-S group showed the biggest risk in all-cause mortality (HR, 2.45; 95% CI, 1.90–3.17; P = 0.00), which suggested high-quality Mediterranean diet might attenuate the risk of all-cause mortality resulting from sedentary behavior. In terms of cox regression analysis of the cardiovascular mortality risk, there was no significant difference in HR between HaMED-non S, MaMED-non S group, LaMED-non S, HaMED-S groups. As for MaMED-S, LaMED-S groups, HR of two groups was progressively increasing and statistically significant. LaMED-S group also shows the biggest risk in cardiovascular mortality (HR, 2.91; 95% CI, 1.77–4.79; P = 0.00). In MetS participants with sedentary behavior, participants with HaMED shows no difference in HR compared to HaMED-non S group while participants with MaMED or LaMED showed increased HR in cardiovascular mortality, which suggested high-quality Mediterranean diet could completely offset the adverse effects of sedentary behavior to cardiovascular mortality (Table 3). Figure 3 visualizes the results of the cox regression analysis. To further verify the reliability of our conclusions, MetS population under another version of definition was used to perform the above statistical analysis (**Appendix 1**). Surprisingly, the similar conclusions were also drawn. The only difference is that in MetS participants without sedentary behavior, LaMED is an independent risk factor for all-cause mortality (HR, 1.27; 95% CI, 1.00–1.60; P = 0.05), which also demonstrated high-quality Mediterranean diet could reduce all-cause mortality in MetS participants without sedentary behavior.

**Table 3:** Cox regression analysis of participants with MetS for all-Cause and cardiovascular mortality according to aMED and sedentary behavior

All-cause Mortality	Model I			Model II			Model III		
	HR (95%CI) *P*			HR (95%CI) *P*			HR (95%CI) *P*		
HaMED-non S	Ref.			Ref.			Ref.		
MaMED-non S	1.07	0.84–1.37	0.57	1.09	0.86–1.4	0.46	1.08	0.79–1.48	0.64
LaMED-non S	**1.33**	**1.09–1.62**	**0.00**	**1.32**	**1.08–1.61**	**0.01**	1.21	0.94–1.56	0.14
HaMED-S	**1.78**	**1.39–2.26**	**0.00**	**1.74**	**1.36–2.21**	**0.00**	**1.55**	**1.15–2.09**	**0.00**
MaMED-S	**1.71**	**1.31–2.23**	**0.00**	**1.71**	**1.31–2.23**	**0.00**	**1.38**	**1.00-1.93**	**0.05**
LaMED-S	**2.43**	**1.97–2.99**	**0.00**	**2.31**	**1.87–2.84**	**0.00**	**2.45**	**1.90–3.17**	**0.00**
**Cardiovascular Mortality**	**Model I**			**Model II**			**Model III**		
	**HR (95%CI)** *** P***			**HR (95%CI)** *** P***			**HR (95%CI)** *** P***		
HaMED-non S	Ref.			Ref.			Ref.		
MaMED-non S	0.92	0.55–1.54	0.74	0.96	0.57–1.60	0.86	1.01	0.53–1.92	0.99
LaMED-non S	**1.55**	**1.04–2.29**	**0.03**	**1.58**	**1.07–2.34**	**0.02**	1.34	0.81–2.21	0.26
HaMED-S	**1.93**	**1.19–3.11**	**0.01**	**1.80**	**1.12–2.91**	**0.02**	1.43	0.78–2.59	0.25
MaMED-S	**2.43**	**1.50–3.95**	**0.00**	**2.52**	**1.55–4.08**	**0.00**	**2.04**	**1.12–3.73**	**0.02**
LaMED-S	**2.91**	**1.94–4.36**	**0.00**	**2.89**	**1.93–4.33**	**0.00**	**2.91**	**1.77–4.79**	**0.00**

HaMED-non S, High aMED without sedentary behavior; MaMED-non S, Medium aMED without sedentary behavior; LaMED-non S, Low aMED without sedentary behavior; HaMED-S, High aMED with sedentary behavior; MaMED-S, Medium aMED with sedentary behavior; LaMED-S, Low aMED with sedentary behavior. Model I was adjusted for age, sex, race, education, marital status and poverty to income ratio; Model II was adjusted for age, sex, race, education, marital status, poverty to income ratio, cardiovascular disease and smoking; Model III was adjusted for age, sex, race, education, marital status, poverty to income ratio, cardiovascular disease, smoking, treatment for hypertension, treatment for diabetes and treatment for cholesterol

HaMED-non S, High aMED without sedentary behavior; MaMED-non S, Medium aMED without sedentary behavior; LaMED-non S, Low aMED without sedentary behavior; HaMED-S, High aMED with sedentary behavior; MaMED-S, Medium aMED with sedentary behavior; LaMED-S, Low aMED with sedentary behavior. Model was adjusted for age, sex, race, education, marital status, poverty to income ratio, cardiovascular disease, smoking, treatment for hypertension, treatment for diabetes and treatment for cholesterol.

### Cox regression analysis of participants with MetS all-cause and cardiovascular mortality in different dietary classes with and without depression

**Fig. 4:**
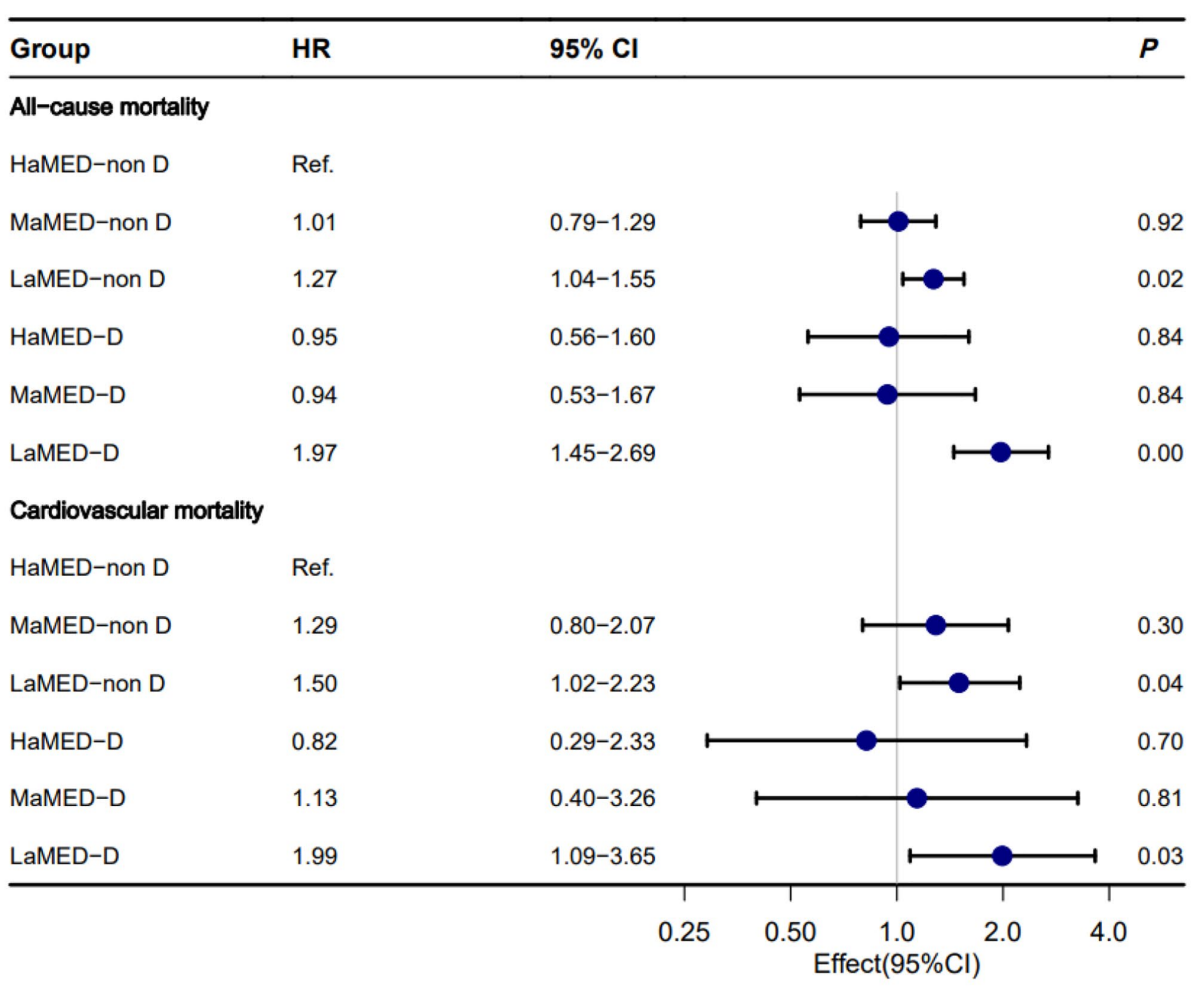
**Forest plot of cox regression analysis of participants with MetS for all-cause and cardiovascular mortality according to aMED and depression**

To investigate the joint effect of the Mediterranean diet and depression on all-cause mortality and cardiovascular mortality in participants with MetS, we divided all paticipants with MetS into the following six groups: HaMED-non D (MetS participants with above median aMED without depression), MaMED-non D (MetS participants with median aMED without depression), LaMED-non D (MetS participants with below median aMED without depression), HaMED-D (MetS participants with above median aMED with depression), MaMED-D (MetS participants with median aMED with depression) and LaMED-D (MetS participants with below median aMED with depression). Cox regression analysis of the all-cause mortality risk for MaMED-non D, LaMED-non D, HaMED-D, MaMED-D, LaMED-D groups using HaMED-non D group as the control group showed that, after the adjustment for age, sex, race, education, marital status, poverty to income ratio, cardiovascular disease, smoking, treatment for hypertension, treatment for diabetes and treatment for cholesterol, there was no significant difference in HR between HaMED-non D group and MaMED-non D group. However, LaMED-non D group showed a increased HR in all-cause mortality (HR, 1.27; 95% CI, 1.04–1.55; P = 0.02) compared to HaMED-non D group, which means in participants with MetS without depression, lower-quality Mediterranean diet significantly increases all-cause mortality. Similar results can still be observed in participants with MetS with depression. LaMED-D group had the highest all-cause mortality (HR, 1.97; 95% CI, 1.45–1.269; P = 0.00). Interestingly, even when MetS participants combined with depression, but with a higher quality Mediterranean diet, all-cause mortality was not significantly increased and was even lower than those without depression but with a lower quality Mediterranean diet. A similar trend can be observed in cardiovascular mortality. Compared to HaMED-non D group, MaMED-non D, HaMED-D and MaMED-D groups did not show significant difference in HR in cardiovascular mortality. Combined low-quality Mediterranean diet shows higher cardiovascular mortality, regardless of depression, in participants with MetS. LaMED-non D group shows the notable risk in cardiovascular mortality (HR, 1.50; 95% CI, 1.02–2.23; P = 0.04) while LaMED-D group shows the biggest risk in cardiovascular mortality (HR, 1.99; 95% CI, 1.09–3.65; P = 0.03) (Table 4). Figure 4 visualizes the results of the cox regression analysis. To further enhance the robustness of the conclusions, we did the same statistical analysis described above in MetS participants defined by another diagnostic criteria. Similar results are presented in **Appendix 2**, which again confirms the reliability of the conclusions.

**Table 4:** Cox regression analysis of participants with MetS for all-cause and cardiovascular mortality according to aMED and depression

All-cause Mortality	Model I			Model II			Model III		
	HR (95%CI) *P*			HR (95%CI) *P*			HR (95%CI) *P*		
HaMED-non D	Ref.			Ref.			Ref.		
MaMED-non D	1.03	0.85–1.25	0.73	1.06	0.87–1.28	0.58	1.01	0.79–1.29	0.92
LaMED-non D	**1.28**	**1.09–1.5**	**0.00**	**1.26**	**1.07–1.48**	**0.00**	**1.27**	**1.04–1.55**	**0.02**
HaMED-D	1.10	0.71–1.70	0.68	0.94	0.60–1.46	0.78	0.95	0.56–1.60	0.84
MaMED-D	1.21	0.78–1.88	0.4	1.1	0.71–1.71	0.68	0.94	0.53–1.67	0.84
LaMED-D	**2.26**	**1.75–2.93**	**0.00**	**1.94**	**1.49–2.52**	**0.00**	**1.97**	**1.45–2.69**	**0.00**
**Cardiovascular Mortality**	**Model I**			**Model II**			**Model III**		
	**HR (95%CI)** *** P***			**HR (95%CI)** *** P***			**HR (95%CI)** *** P***		
HaMED-non D	Ref.			Ref.			Ref.		
MaMED-non D	1.11	0.76–1.63	0.58	1.19	0.81–1.74	0.38	1.29	0.80–2.07	0.30
LaMED-non D	**1.53**	**1.12–2.08**	**0.01**	**1.54**	**1.14–2.10**	**0.01**	**1.50**	**1.02–2.23**	**0.04**
HaMED-D	1.44	0.65–3.17	0.37	1.16	0.52–2.56	0.71	0.82	0.29–2.33	0.70
MaMED-D	1.37	0.58–3.19	0.47	1.24	0.53–2.91	0.62	1.14	0.40–3.26	0.81
LaMED-D	**2.25**	**1.34–3.8**	**0.00**	**1.87**	**1.11–3.17**	**0.02**	**1.99**	**1.09–3.65**	**0.03**

HaMED-non D, High aMED without depression; MaMED-non D, Medium aMED without depression; LaMED-non D, Low aMED without depression; HaMED-D, High aMED with depression; MaMED-D, Medium aMED with depression; LaMED-D, Low aMED with depression. Model I was adjusted for age, sex, race, education, marital status and poverty to income ratio; Model II was adjusted for age, sex, race, education, marital status, poverty to income ratio, cardiovascular disease and smoking; Model III was adjusted for age, sex, race, education, marital status, poverty to income ratio, cardiovascular disease, smoking, treatment for hypertension, treatment for diabetes and treatment for cholesterol.

HaMED-non D, High aMED without depression; MaMED-non D, Medium aMED without depression; LaMED-non D, Low aMED without depression; HaMED-D, High aMED with depression; MaMED-D, Medium aMED with depression; LaMED-D, Low aMED with depression. Model was adjusted for age, sex, race, education, marital status, poverty to income ratio, cardiovascular disease, smoking, treatment for hypertension, treatment for diabetes and treatment for cholesterol.

### Associations of individual food components of the Mediterranean diet with all-cause and cardiovascular in NHANES participants with MetS

To examine in depth the association between Mediterranean diet components and all-cause mortality and cardiovascular mortality in MetS participants, cox regression analysis was utilized. Among the components of the MED diet, greater vegetables, legumes, nuts intake and high MUFA/SFA ratio were significantly associated with lower all-cause mortality after adjustment for age, sex, race, education, marital status, poverty to income ratio, cardiovascular disease, smoking, treatment for hypertension, treatment for diabetes and treatment for cholesterol in participants with MetS (Table 5). Greater vegetables intake was significantly associated with lower cardiovascular mortality after adjustment for age, sex, race, education, marital status, poverty to income ratio, cardiovascular disease, smoking, treatment for hypertension, treatment for diabetes and treatment for cholesterol in participants with MetS while more red/processed meat intake was significantly associated with higher cardiovascular mortality. Similar results were also shown for another diagnostic criterion of MetS (**Appendix 3**). One difference is that under this version of the criteria, greater vegetable intake does not significantly reduce cardiovascular mortality in participants with MetS.

**Table 5:** Associations between Mediterranean diet components and all-cause and cardiovascular mortality in participants with MetS

	All-cause Mortality			Cardiovascular Mortality		
	HR (95%CI) *P*			HR (95%CI) *P*		
Vegetables	**0.89**	**0.80–0.99**	**0.04**	**0.81**	**0.65-1.00**	**0.05**
Fruits	0.96	0.89–1.05	0.36	0.95	0.81–1.12	0.56
Grain	0.99	0.96–1.02	0.43	0.98	0.93–1.05	0.61
Meat	1.00	0.96–1.05	0.87	**1.07**	**1.00-1.15**	**0.04**
Legumes	**0.91**	**0.84–0.98**	**0.02**	0.93	0.81–1.08	0.34
Seafood	0.96	0.91–1.02	0.23	0.88	0.77–1.01	0.08
Nuts	**0.90**	**0.83–0.97**	**0.01**	0.89	0.75–1.04	0.14
MUFA/SFA	**0.75**	**0.58–0.98**	**0.03**	0.80	0.48–1.32	0.38
Alcohol	1.00	1.00–1.00	0.67	0.99	0.97-1.00	0.07

Model was adjusted for age, sex, race, education, marital status, poverty to income ratio, cardiovascular disease, smoking, treatment for hypertension, treatment for diabetes and treatment for cholesterol.

## Discussion

In our study, we discovered that the Mediterranean diet was a strong protective factor for mortality outcomes in people with MetS, whereas depression and sedentary behavior were significant risk factors. The health advantages of a Mediterranean diet are abundantly established. First, patients with diabetes may have altered lipid metabolism and IR due to unsaturated fatty acids in the Mediterranean diet. According to a randomized controlled trial, patients with T2D who follow a diet typical of Mediterranean nations may have a decrease in all-cause and CVD-related mortality [25]. Olive oil, nuts, and seeds from plant-based diets are rich in monounsaturated fatty acids (MUFAs) and polyunsaturated fatty acids (PUFAs), which may be responsible for improvements in glucose metabolism, insulin sensitivity, lipids, and CVD risk in diabetics. The anti-diabetic effects of these nutrients were supported by a meta-analysis of 24 randomized controlled trials [25], which also showed that a high MUFAs or high PUFAs plant-based diet is superior to a low-fat, high-carbohydrate diet for glycemic control, lipid profile, and systolic blood pressure in diabetic patients. According to a number of in vitro studies, flavonoids may have potent inhibitory activity against intestinal -glucosidase, the enzyme that catalyzes the cleavage of glucose from disaccharides, delaying glucose absorption and reducing postprandial hyperinsulinemia and hyperglycemia [27]. It is also more abundant in extra virgin olive oil and has a higher bioavailability [28]. An experimental research showed that oleacein’s ability to prevent mice from HFD-induced obesity is mediated through the modification of adipogenesis regulators. Peripheral insulin sensitivity is effectively increased by protection against HFD-induced obesity [29]. Numerous epidemiological and clinical research have found that taking green tea supplements significantly reduces the risk of developing chronic diseases [30]. Endothelial dysfunction, according to current knowledge, is linked to oxidative stress, decreased nitric oxide bioavailability, increased anticoagulant properties and platelet aggregation, increased expression of adhesion molecules (such as P- and E-selectin, intercellular adhesion molecule-1 (ICAM-1), and leukocyte adhesion molecules, such as vascular cell adhesion molecule-1 (VCAM-1), increased expression of pro-inflammatory chemokines (i.e. IL-1b.)) [31]. By blocking nuclear factor-kB, polyphenols directly target and reduce the expression of cytokines, chemokines, and adhesion molecules brought on by inflammatory stimuli in the human endothelium system in vitro (NF-kB) signal pathway [32]. IR, glucose homeostasis, lipid metabolism, and atherogenesis all include HMGA1, which has recently been shown as a strong molecular link between two overlapping clinical characteristics (i.e. T2D and CVD). Through hmga1-mediated processes, plant-derived polyphenols can prevent or reverse saturated fatty acid-induced IR [33].

Since MetS and depression are both risk factors for cardiovascular disease, their connection has received a lot of attention recently. An elevated risk of developing MetS among middle-aged adult university graduates from a Spanish cohort was linked to depression in a prospective cohort study [34]. A recent two-sample bi-direction al Mendelian randomization study [35] demonstrated that for MetS and its components on the risk of depression, no causal connections were found. Depression is a risk factor for MetS and its components, according to the current MR research (waist circumference, hypertension, FBG, triglycerides, and HDL-C). In order to effectively treat MetS and its components, early depression detection and prevention are essential. The most significant potential targets for preventative interventions in men and women, respectively, emerged as hypertriglyceridemia and unhealthy waist circumference [36]. A cross-sectional survey of 13,626 US adults offer some evidence that eating chocolate, especially dark chocolate, may lower the risk of clinically significant depressive symptoms [37]. In a healthy young population, regular eating of peanuts and peanut butter may improve memory performance and stress reactivity. These outcomes appear to be connected to consumption of peanut polyphenols and elevated fecal SCFA levels [38]. Arab et al. found when compared to non-nut users, depression scores were considerably lower among nut consumers, and especially walnut consumers [39]. Because the numerous nutrients in the Mediterranean diet can alleviate the chronic inflammation that depression causes in the body, it became clear from our study that a high-quality Mediterranean diet can really reverse the detrimental consequences of depression.

Studying the risk and protective factors linked to MetS is crucial because of the rising prevalence of MetS and the socioeconomic cost it imposes on society. Already, the MetS is an independent risk factor for cardiovascular disease and death. However, poor lifestyle choices, such as inactivity, and sedentary behavior are frequently linked to MetS patients. The MetS in conjunction with these other risk factors can hasten the incidence of adverse events. It is generally known that exercise reduces IR in population with MetS [40]. Both observational and interventional research point to a critical role for increased fitness and physical activity in reducing the risk of the MetS. Interventions that include physical activity have a positive impact on each aspect of the MetS to varying degrees [41]. In our investigation, the quantity and the intensity of exercise was significantly related to mortality in the population with MetS. There is no doubt that exercise is an effective means of improving MetS.

Sedentary behavior, while still a reliable indicator of the quality of exercise, was clearly a risk factor in our study. At the same time being sedentary tends to mean lower levels of exercise. A national study showed that sedentary behaviors in the US population either increased from 2001 to 2016 or remained generally stable and high [42]. Besides, Healy et al. found the deleterious relationships between long periods of inactivity and inflammatory and metabolic indicators and reducing and breaking up sedentary time through clinical communications and preventive health messages may reduce the risk of cardiovascular disease [43]. Another study found that sedentary time was associated with the prognosis of cancer patients. The highest risks of death from all causes and cancer were related with the combination of prolonged sitting with lack of physical activity, which was extremely common [44]. There are many studies on the poor prognosis due to sedentary behavior, which often produces undesirable myocardial metabolites and further causes metabolic disorders [45–47]. People with MetS and sedentary behavior had a considerably greater mortality rate than those with MetS alone. A high-quality Mediterranean diet demonstrated a protective impact in both groups. However, a high-quality Mediterranean diet considerably reduces the negative consequences of sedentary behavior in patients with MetS who also have other sedentary behavior, but it does not totally eradicate them. Therefore, the World Health Organization strongly advises that all people engage in 150–300 min per week of moderate intensity exercise, 75–150 min per week of strenuous intensity exercise, or some equal combination of moderate intensity and vigorous intensity aerobic exercise. Reduced sedentary behavior is also advised for people of all ages and abilities [21].

## Limitation

Our study suffers from the following shortcomings: First, in order to make the findings more robust, we did cox regression analysis by adjusting for a series of covariates. However, the information on these covariates was collected by the NHANES questionnaire, and a number of these variables may have been missing, which further led to a reduction in included population of this study. Secondly, dietary data was collected by questionnaire. Although NHANES interviewers were trained to perform better documentation of diet and we used the average of dietary data from two different weeks to minimize bias in statistical process, there may be some discrepancies between participants’ dietary habits and the questionnaire. Last, there is a minor flaw in the patient mortality follow-up data. Deaths due to pre-existing serious illnesses of participants may potentially contribute to contamination of mortality data.

## Conclusion

In this study, we found that participants with MetS with adherence to high-quality or moderate-quality Mediterranean diet were significantly associated with lower all-cause mortality as well as cardiovascular mortality during the follow-up period in the NHANES study. High-quality or moderate-quality Mediterranean diet could attenuate, even reverse the adverse effects of sedentary behavior and depression on all-cause and cardiovascular mortality in participants with MetS. Among the components of the MED diet, greater intakes of vegetables, legumes, nuts and high MUFA/SFA ratio were significantly associated with lower all-cause mortality and greater vegetables intake was significantly associated with lower cardiovascular mortality, while more red/processed meat intake was significantly associated with higher cardiovascular mortality in participants with MetS.

## Data Availability

The original data presented in the study are included in the article. Further inquiries can be directed to the corresponding authors.

## Appendix 1 Cox regression analysis of participants with MetS (Another diagnostic criteria) for all-cause and cardiovascular mortality according to aMED and sedentary behavior

**Table:**

All-cause Mortality	Model I			Model II			Model III		
	HR (95%CI) P			HR (95%CI) P			HR (95%CI) P		
HaMED-non S	Ref.			Ref.			Ref.		
MaMED-non S	1.10	0.89–1.37	0.37	1.12	0.9–1.39	0.32	1.18	0.89–1.57	0.25
LaMED-non S	**1.37**	**1.14–1.64**	**0.00**	**1.35**	**1.13–1.62**	**0.00**	**1.27**	**1.00-1.60**	**0.05**
HaMED-S	**1.78**	**1.43–2.23**	**0.00**	**1.74**	**1.39–2.17**	**0.00**	**1.65**	**1.26–2.17**	**0.00**
MaMED-S	**1.72**	**1.34–2.19**	**0.00**	**1.70**	**1.33–2.17**	**0.00**	**1.54**	**1.14–2.08**	**0.00**
LaMED-S	**2.46**	**2.03–2.98**	**0.00**	**2.33**	**1.93–2.83**	**0.00**	**3.67**	**2.38–5.66**	**0.00**
**Cardiovascular Mortality**	**Model I**			**Model II**			**Model III**		
	**HR (95%CI) P**			**HR (95%CI) P**			**HR (95%CI) P**		
HaMED-non S	Ref.			Ref.			Ref.		
MaMED-non S	1.00	0.64–1.57	1.00	1.05	0.67–1.65	0.82	1.34	0.77–2.34	0.29
LaMED-non S	**1.44**	**1.01–2.05**	**0.04**	**1.43**	**1.00-2.04**	**0.05**	1.28	0.81–2.03	0.30
HaMED-S	**1.80**	**1.16–2.78**	**0.01**	**1.66**	**1.07–2.56**	**0.02**	1.50	0.88–2.58	0.14
MaMED-S	**2.20**	**1.40–3.44**	**0.00**	**2.23**	**1.42–3.49**	**0.00**	**2.06**	**1.18–3.59**	**0.01**
LaMED-S	**2.78**	**1.92–4.01**	**0.00**	**2.60**	**1.80–3.76**	**0.00**	**2.75**	**1.74–4.35**	**0.00**

HaMED-non S, High aMED without sedentary behavior; MaMED-non S, Medium aMED without sedentary behavior; LaMED-non S, Low aMED without sedentary behavior; HaMED-S, High aMED with sedentary behavior; MaMED-S, Medium aMED with sedentary behavior; LaMED-S, Low aMED with sedentary behavior. Model I was adjusted for age, sex, race, education, marital status and poverty to income ratio; Model II was adjusted for age, sex, race, education, marital status, poverty to income ratio, cardiovascular disease and smoking; Model III was adjusted for age, sex, race, education, marital status, poverty to income ratio, cardiovascular disease, smoking, treatment for hypertension, treatment for diabetes and treatment for cholesterol.

## Appendix 2 Cox regression analysis of participants with MetS (Another diagnostic criteria) for all-cause and cardiovascular mortality according to aMED and depression

**Table:**

All-cause Mortality	Model I			Model II			Model III		
	HR (95%CI) *P*			HR (95%CI) *P*			HR (95%CI) *P*		
HaMED-non D	Ref.			Ref.			Ref.		
MaMED-non D	1.07	0.90–1.28	0.43	1.08	0.91–1.29	0.37	1.10	0.89–1.37	0.37
LaMED-non D	**1.33**	**1.15–1.54**	**0.00**	**1.31**	**1.13–1.52**	**0.00**	**1.31**	**1.09–1.57**	**0.00**
HaMED-D	1.25	0.84–1.85	0.27	1.07	0.72–1.59	0.73	1.02	0.63–1.65	0.93
MaMED-D	1.13	0.74–1.74	0.57	1.03	0.67–1.59	0.88	0.88	0.51–1.52	0.64
LaMED-D	**2.23**	**1.74–2.85**	**0.00**	**1.92**	**1.5–2.46**	**0.00**	**1.91**	**1.42–2.55**	**0.00**
**Cardiovascular Mortality**	**Model I**			**Model II**			**Model III**		
	**HR (95%CI)** *** P***			**HR (95%CI)** *** P***			**HR (95%CI)** *** P***		
HaMED-non D	Ref.			Ref.			Ref.		
MaMED-non D	1.15	0.81–1.62	0.44	1.22	0.86–1.72	0.26	1.4	0.92–2.13	0.12
LaMED-non D	**1.51**	**1.14–2.01**	**0.00**	**1.51**	**1.14–2.01**	**0.00**	**1.44**	**1.01–2.05**	**0.05**
HaMED-D	1.65	0.82–3.33	0.16	1.30	0.64–2.63	0.47	0.91	0.36–2.34	0.85
MaMED-D	1.40	0.64–3.07	0.40	1.27	0.58–2.8	0.55	1.25	0.49–3.22	0.64
LaMED-D	**2.18**	**1.33–3.58**	**0.00**	**1.78**	**1.08–2.92**	**0.02**	**1.93**	**1.10–3.39**	**0.02**

HaMED-non D, High aMED without depression; MaMED-non D, Medium aMED without depression; LaMED-non D, Low aMED without depression; HaMED-D, High aMED with depression; MaMED-D, Medium aMED with depression; LaMED-D, Low aMED with depression. Model I was adjusted for age, sex, race, education, marital status and poverty to income ratio; Model II was adjusted for age, sex, race, education, marital status, poverty to income ratio, cardiovascular disease and smoking; Model III was adjusted for age, sex, race, education, marital status, poverty to income ratio, cardiovascular disease, smoking, treatment for hypertension, treatment for diabetes and treatment for cholesterol.

## Appendix 3 Associations between Mediterranean diet components and all-cause and cardiovascular mortality in participants with MetS (Another diagnostic criteria)

**Table:**

	All-cause Mortality			Cardiovascular Mortality		
	HR (95%CI) *P*			HR (95%CI) *P*		
Vegetables	**0.87**	**0.79–0.96**	**0.01**	0.85	0.70–1.03	0.09
Fruits	0.95	0.88–1.02	0.17	0.90	0.80–1.02	0.10
Grain	0.98	0.95–1.01	0.23	0.99	0.94–1.04	0.70
Meat	1.01	0.98–1.05	0.50	**1.07**	**1.01–1.14**	**0.03**
Legumes	**0.92**	**0.86–0.98**	**0.01**	0.94	0.83–1.06	0.32
Seafood	0.97	0.91–1.02	0.21	0.91	0.78–1.05	0.20
Nuts	**0.91**	**0.85–0.98**	**0.01**	0.96	0.86–1.08	0.53
MUFA/SFA	**0.78**	**0.61–0.99**	**0.04**	0.83	0.53–1.31	0.43
Alcohol	1.00	1.00-1.01	0.52	0.99	0.98-1.00	0.09

Model was adjusted for age, sex, race, education, marital status, poverty to income ratio, cardiovascular disease, smoking, treatment for hypertension, treatment for diabetes and treatment for cholesterol.
